# Seroprevalence of *Toxoplasma gondii* and associated risk factors among HIV-infected women within reproductive age group at Mizan Aman General Hospital, Southwest Ethiopia: a cross sectional study

**DOI:** 10.1186/s13104-017-2390-6

**Published:** 2017-01-26

**Authors:** Ayalew Jejaw Zeleke, Yayehirad Alemu Melsew

**Affiliations:** 1 Department of Parasitology, School of Biomedical and Laboratory Science, College of Medicine and Health Sciences, University of Gondar, Gondar, Ethiopia; 2 Department of Epidemiology and Biostatistics, Institute of Public Health, College of Medicine and Health Sciences, University of Gondar, Gondar, Ethiopia

**Keywords:** Seroprevalence, *Toxoplasma gondii*, HIV-infected women, Ethiopia

## Abstract

**Background:**

Toxoplasmosis is serious in the case of immune suppression and prenatal transmission. In immunocompromised hosts, it is manifested primarily as a life-threatening condition, toxoplasmic encephalitis. Congenital toxoplasmosis results in abortion or congenitally acquired disorders which primarily affect the central nervous system. This study assessed seroprevalence of *Toxoplasma gondii* (*T. gondii*) infection and associated factors among HIV-infected women within the reproductive age group (18–49 years) at Mizan Aman General Hospital, Southwest Ethiopia. An institution based cross-sectional study was conducted from February 01 to May 30, 2015. Systematic random sampling technique was employed for participant selection. Enzyme linked immuno sorbent assay was used to test for *T. gondii* from venous blood specimens. Participants were interviewed using structured questionnaire for different variables. Descriptive statistics, binary and multivariable logistic regression analyses were performed during data analysis. *P* value of less than 0.05 was considered statistically significant.

**Results:**

A total of 270 HIV-infected women within the reproductive age group were included in the study. Mean age of the respondents was 31 years (SD = ±6.5). Of the total study participants, 255 (94.4%), 95% CI (91.6, 97.2%) were found to be seropositive for *T. gondii* anti-immunoglobulin G (IgG) antibody, and 6 (2.2%), 95% CI (1.3, 3.1%) for anti-immunoglobulin M (IgM). All the anti-IgM positive samples were also positive for IgG. Multivariate analysis showed that; age within 28–37 years (Adjusted Odds Ratio [AOR] 2.58, 95% CI 1.01, 6.60), level of education with unable or only able to read and write (AOR = 4.46, 95% CI 1.20, 16.60), and substance abuse (AOR = 4.49, 95 CI 1.60, 12.55) were significantly associated with seropositivity of *T. gondii* infection.

**Conclusions:**

Seroprevalence of toxoplasmosis among the HIV-infected women in the childbearing age group in Mizan Aman was high. Age, educational status and drug addiction were identified as factors associated with *T. gondii* infection. Education of HIV-infected women about the transmission and prevention methods of *T. gondii* infection is important. Besides, studies on incidence of toxoplasmosis in newborns and infants are recommended.

## Background

Toxoplasmosis is a globally distributed zoonosis [1] caused by the ubiquitous obligatory intracellular coccidian protozoan organism, *T. gondii* that infects a wide range of animals, humans and birds. It is an opportunistic parasitic infection in immunocompromised hosts and is a major public health concern as the disease is serious in terms of mortality or physical and/or psychological sequelae in patients with HIV disease [2].

In the vast majority of immunocompetent human hosts, *T. gondii* ensue a latent infection characterized by the persistence of the organism in tissues (primarily brain, skeletal muscles, and heart) without causing disease [3]. However, in chronically infected individuals who develop defects incell-mediated immunity a symptomatic disease more likely occurs as a result of reactivation of latent infection if their CD4+T cell count decreases below 200 cells/μL. Toxoplasmosis among acquired immune deficiency Syndrome (AIDS) patients manifests primarily as a life threatening condition, toxoplasmic encephalitis (TE). That is accompanied by headache, disorientation, drowsiness, hemiparesis, reflex changes, and convulsions. Coma and death may ensue. Toxoplasmosis-HIV co-infected patients have 30–40% risk of developing toxoplasmic encephalitis [4].

Congenital toxoplasmosis may result in women infected by the parasite during pregnancy, or in women co-infection with HIV. Thus, HIV and pregnancy may strengthen its incapacitating impact. Global estimates of maternal–fetal transmission are approximately 20–33% of newly infected mothers. Important variations have also been observed according to gestational age at the time of maternal infection, with transmission rates increasing as the pregnant women approach to birth. Congenital infection may result in a higher risk of tragic outcomes such as retinochoroiditis/visual impairment, hydrocephalus, convulsions, intracerebral calcifications (intellectual impairment), and abortion [5]. The harmful effects of *T. gondii* in immunocompromised individuals suggest screening of HIV-infected women for toxoplasmosis to minimize the deleterious effect of the infection.

Diagnosis of toxoplasmosis commonly involves detection of IgM and IgG antibodies in the patient’s serum. In the course of *T. gondii* infection, IgM antibodies are detected within a few days to one week of infection. The IgG antibodies are detected within 1–2 weeks of infection, reaching peak after four months, and then declining to lower levels and remaining positive for the remainder of the individual’s life. A negative IgM antibody test essentially excludes acute infection while a positive IgG test with a negative IgM indicates chronic infection [6]. Therefore, detection and monitoring of anti-*T. gondii* antibodies are of a great important in HIV-infected patients.

Ethiopia is one of the countries with the highest HIV epidemic with approximately 1.5 million people being infected with HIV/AIDS or living with AIDS [7]. Several people in Benchi Maji zone are HIV-infected, with higher incidence among women with in the reproductive age group (*Zonal health office report, unpublished*). In spite of the higher rate of HIV infection among the women, and the presence of stray cats as well as suitable climatic conditions favouring survival of the parasite in the study area, to our knowledge, there is no documented data on the epidemiology of toxoplasmosis among HIV-infected women within the reproductive age group. Besides this, *T. gondii* screening of women in general and those living with HIV in particular is yet not practiced as an antenatal examination in the area. Therefore, this study was aimed at determining seroprevalence of *T. gondii* and associated factors among HIV-infected women within reproductive age group attending Mizan Aman general Hospital antiretroviral treatment (ART) clinic, which will help as a base-line data and provides information for evidence-based decision.

## Methods

### Study setting

The study was conducted at Mizan-Aman General Hospital from February 01 to May 30, 2015. The hospital is located 566 kms southwest of Addis Ababa. The area is characterized by humid warm climate (mean annual temperature ranges between 15.1 and 27 °C), perennial rivers, and is considered ideal for agriculture and human settlement. The mean annual rainfall ranges from 400 to 2000 mm. The major economic activity of the urban inhabitants is trading while subsistence farming is the dominant means of earning a living for the surrounding rural communities. The hospital was established in 1971. Currently, the hospital serves an estimated 2 million people in Mizan Aman town and its surroundings.

### Study design and sample size determination

Institution based cross-sectional study was conducted. The minimum sample size was calculated using single population proportion formula n = (Z_1−α/2_)^2^p (1 − p)/d^2^, with the following assumptions: prevalence (p) of 80% from a previous study [8–10], 95% confidence level, 5% margin of error, and 10% for anticipated non-response rate. Accordingly, the minimum sample size (n) was found to be 271 HIV-infected women within reproductive age group. During participant selection, the number HIV-infected women within reproductive age group in the study period was first estimated by taking the monthly average number of clients attending ART clinic in the previous four months (October 01/2014–January 30/2015). Then, the number of women enrolled was allocated proportionally to each week according to the total number of clients proposed to be attended during the study period. Depending on this analysis an approximate numbers of women who were expected to attend ART service during the three months of study period were determined. Finally, systematic random sampling was used to select study participants.

### Survey of demographic and clinical profile, and personal risk factors

Data on demographic profile of the participants and factors predisposing to *T. gondii* infection were gathered using pretested questionnaire. The questions included information on age, educational status, having contact with domestic cats, substance abuse, hand washing practices, source of drinking water, habit of eating raw meat and vegetables. The clinical profiles of the study participants assessed include abortion, child with visual impairment, pregnancy status, ART status, child with mental retardation, child with epilepsy, blood transfusion, and recent CD4 count. Clinical nurses who were working in the ART clinic and fluent with the local languages (Amharic and Bench) interviewed the study participants.

### Serological assays

Five millilitres of venous blood was aseptically collected into plain tube at the ART laboratory by senior laboratory technician. The collected blood samples were allowed to stand for an hour within the test tubes on a table and the sera were separated and stored at −20 °C until completion of data collection. Finally, the samples were transported with ice box to Jimma Blood Bank Laboratory for detecting anti-*T. gondii* IgM and IgG antibodies using ELISA. The serum samples were screened for anti-*T. gondii* IgG and IgM antibodies using the ELISA “Toxo IgG and Toxo IgM µ-Capture” kit (*HUMAN, GmbH.65205 Wiesbaden*-*Biochemical Diagnostic, Germany*) according to the manufacturer’s instructions. Positive and negative controls were used with each series of anti *T. gondii* IgG/IgM test; results were obtained by reading the optical density at 450 nm using spectrophotometer and values were interpreted comparing with the cut-off value.

### Data processing and analysis

Data were checked for completeness, coded and entered into computer. The data were cleaned and analyzed by using SPSS-20 software. Descriptive statistics were computed to describe the study participants with different variables and displayed with tables and graphs. Both bivariate and multivariable logistic regression models were fitted to identify associated risk factors. *P* values less than 0.05 were considered statistically significant.

## Results

### Socio-demographic characteristics

A total of 270 women within reproductive age group (18–49 years) were included in the study giving response rate of 99.6%. The mean age of respondents was 31 years (SD = ±6.5). The highest proportion of respondents were Orthodox Christian in religion comprising 160 (59.3%) and 148 (54.8%) of the respondents had level of education with only primary education (Table 1).

**Table 1:** Socio-demographic characteristics of HIV-infected women in the childbearing age group attending ART clinic at Mizan-Aman General Hospital, Southwest Ethiopia, May 2015

Variables	Numbers	%
*Age (years)*		
18–27	84	95.5
28–37	124	94.7
38–49	47	92.2
*Religion*		
Orthodox Christian	160	59.3
Protestant	44	16.3
Muslim	65	24.1
Other	1	0.4
*Occupation*		
Housewife	103	38.1
Merchant	92	34.1
Student	5	1.9
Government employee	25	9.3
Farmer	45	16.7
*Level of education*		
Unable to read and write	59	21.9
Only able to read and write	8	3.0
Primary school	148	54.8
High school	39	14.4
College or university graduate	16	5.9
*Residence*		
Urban	203	75.2
Rural	67	24.8

### Seroprevalence of *T. gondii*

Anti-*T. gondii* Immunoglobulin G (IgG) was found in the serum sample of 255 (94.4%) women while Immunoglobulin M (IgM) was found only in 6 (2.2%) serum samples (Table 2).

**Table 2:** Seroprevalence of *T. gondii* with respect to socio-demographic characteristics at Mizan Aman General Hospital, Southwest Ethiopia, May 2015

Variables	IgG		IgM	
	Positive	Negative	Positive	Negative
*Age*				
18–27	84 (95.5%)	4 (4.5%)	4 (4.5%)	84 (95.5%)
28–37	124 (94.7%)	7 (5.3%)	2 (1.5%)	129 (98.5%)
38–49	47 (92.2%)	4 (7.8%)	0 (0.0%)	51 (100%)
Total	255 (94.4%)	15 (5.5%)	6 (2.2%)	97.8%
*Ever had contact with cat*				
Yes	25 (92.6%)	2 (7.4%)	1 (3.7%)	26 (96.3%)
No	230 (94.7%)	13 (5.3%)	5 (2.1%)	238 (97.9%)
*Educational level*				
Unable to/only/read and write	64 (95.5%)	3 (4.5%)	0 (0.0%)	67 (100%)
Primary education	140 (94.6%)	8 (5.4%)	3 (2%)	145 (98%)
High school and above	51 (92.7%)	4 (7.3%)	3 (5.5%)	52 (94.5%)
*Ever eaten uncooked meat*				
Yes	66 (98.5%)	1 (1.5%)	1 (1.5%)	66 (98.5%)
No	189 (93.1%)	14 (6.9%)	5 (2.5%)	198 (97.5%)
*Have addiction (alcohol, cigarette, Khat)*				
Yes	147 (96.7%)	5 (3.3%)	5 (3.3%)	147 (96.7%)
No	108 (91.5%)	10 (8.5%)	1 (0.8%)	117 (99.2%)
*Ever had blood transfusion*				
Yes	19 (95%)	1 (5%)	0	20 (100%)
No	236 (94.4%)	14 (5.6%)	6 (2.4%)	244 (97.6%)
*Have child with visual impairment*				
Yes	2 (100%)	0	0	2 (100%)
No	253 (94.4%)	15 (5.6%)	6 (2.2%)	262 (97.8%)
*Have child with epilepsy*				
Yes	3 (100%)	0	0	3 (100%)
No	252 (94.4%)	15 (5.6%)	6 (2.2%)	261 (97.8%)

### Behavioural characteristics

The behavioural characteristics of studied population were assessed. Accordingly, only 27 (10%) responded that they had contact with cat while more than half of the women 152 (56.3%) expressed that they had addiction to one or more of substances (Khat chewing, alcohol and/or cigarette smoking) (Table 3).

**Table 3:** Behavioural characteristics HIV infected women at Mizan Aman General Hospital, Ethiopia, 2015

Variables	Numbers	%
*Do you always wash your hands before meal*		
Yes	219	81.1
No	51	18.9
*Have you ever contact with cat?*		
Yes	27	10.0
No	243	90.0
*Have you ever eaten uncooked vegetables?*		
Yes	120	44.4
No	150	55.6
*Have you ever eaten uncooked meat?*		
Yes	67	24.8
No	203	75.2
*Where do you get drinking water from?*		
Pond	8	3.0
Well	45	16.7
Spring	19	7.0
Pipe	198	73.3
*Drug addiction (Chat, alcohol, etc.)*		
Yes	152	56.3
No	118	43.7

### Clinical characteristics

At the time of data collection 14 (5.2%) women were pregnant. Majority of respondents 235 (87%) were on Highly Active Antiretroviral therapy (HAART). And also, 111 (41.2%) and 112 (41.5%) had CD4 count between 200–499 and ≥500 cells/m^3^, respectively (Table 4).

**Table 4:** Clinical characteristics of HIV infected women at Mizan Aman General Hospital, Ethiopia, 2015

Variables	Numbers	%
*Have you ever received blood transfusion?*		
Yes	20	7.4
No	250	92.6
*Current pregnancy status*		
Pregnant	14	5.2
Not Pregnant	256	94.8
*Have you ever had abortion*?		
Yes	70	25.9
No	200	74.1
*Child with visual impairment*		
Yes	2	0.7
No	268	99.3
*Child with mental retardation*		
Yes	6	2.2
No	264	97.8
*Child with epilepsy*		
Yes	3	1.1
No	267	98.9
*ART status*		
With HAART	235	87.0
Without HAART	35	13.0
*Recent CD4 count*		
<100	12	4.4
100–199	35	13.0
200–499	111	41.1
>500	112	41.5

### Factors associated with seroprevalence of *T. gondii*

The bivariate binary logistic regression model was fitted for all explanatory variables and ten variables; age, educational level, occupation, hand washing habit before meal, contact with cat, eating uncooked vegetable and meat, drug addiction, unsafe drinking water and history of abortion were found to be significantly associated with seroprevalence of *T. gondii*.

Under multivariate logistic regression analysis only three variables (age, educational level and drug addiction) were found to be significantly associated with seroprevalence of *T. gondii* (Table 5). Accordingly, women with age between 28 and 37 years were 2.6 times more likely to be seropositive compared to whose age was between 18 and 27 years [AOR = 2.58, 95% CI 1.01,6.60]. As far as educational status was concerned, women who were unable or only able to read and write were 4.5 times at higher risk of being seropositive than women with high school and above level of education [AOR = 4.46, 95% CI 1.20, 16.60]. Similarly, women with primary education were 3.6 times more likely to be seropositive compared to women of high school and above [AOR = 3.56, 95% CI 1.48, 8.56]. The other explanatory variable which was found to be significantly associated with seroprevalence of *T. gondii* was substance abuse. Accordingly, women who had drug addiction were 4.9 times at higher risk of *T. gondii* than those who did not have substance abuse [AOR = 4.49, 95 CI 1.60, 12.55].

**Table 5:** Factors associated with toxoplasmosis among HIV infected women at Mizan Aman General Hospital, Ethiopia, 2015

Variable	Sero-status		Crude OR [95% CI]	Adjusted OR [95% CI]
	Positive	Negative		
*Age*				
18–27	84	4	1	1
28–37	124	7	17.71 [8.27, 37.93]*	2.57 [1.00, 6.59]*
38–49	47	4	11.75 [4.23, 32.61]*	1.93 [0.58, 6.31]
*Educational level*				
Only read & write/below	64	3	21.3 [6.70, 67.90]*	4.46 [1.2, 16.59]*
Primary school	140	8	17.5 [8.58, 35.68]*	3.56 [1.481, 8.56]*
High school and above	51	4	1	1
*Occupation*				
Housewife	100	3	1	1
Merchant	86	6	14.33 [6.26, 32.79]*	
Government employee	26	4	6.5 [2.26, 18.62]*	
Farmer	43	2	21.5 [5.20, 88.75]*	
*Hand wash before meal*				
Yes	206	13	1	1
No	49	2	24.5 [5.95, 100.74]*	3.26 [0.71, 15.03]
*Contact with cat*				
Yes	25	2	1	
No	230	13	17.692 [10.119, 30.935]*	
*Eat uncooked vegetable*				
Yes	113	7	16.14 [7.52, 34.63]*	
No	142	8	1	
*Eat uncooked meat*				
Yes	66	1	66 [9.16, 475.521]*	6.61 [0.84, 51.50]
No	189	14	1	1
*Drug addiction*				
Yes	147	5	29.4 [12.05, 71.68]*	4.48 [1.60, 12.55]*
No	108	10	1	
*Drinking water source*				
Unsafe water	69	3	23 [7.24, 73.07]*	
Safe water	186	12	1	
*History of abortion*				
Yes	63	7	9 [4.12,19.65]*	
No	192	8	1	

*OR* odd ratio

* Found significant at 0.05 level significance

## Discussion

This study assessed seroprevalence of *T. gondii* infection and associated risk factors among the HIV-infected women in the childbearing age group in Mizan Aman General Hospital, Southwest Ethiopia.

In this study, seroprevalence of anti-*T. gondii* IgG (94.4%) & IgM (2.2%) indicate that *T. gondii* infection is endemic in the studied population. Primary infection with *T. gondii* results in initial IgM anti-*T. gondii* antibody response, followed by IgG antibody, remains detectable for life. Detection of IgG anti-*T. gondii* antibody, therefore, indicates chronic infection with the parasite. Chronic *T. gondii* infection in HIV-infected individuals is a risk for development of cerebral toxoplasmosis [11], and congenitally transmitting the infection to the fetus in HIV-infected women [5], especially when CD4+T lymphocyte count falls below 100 cells/L [12].

The high prevalence of *T. gondii* infection in the HIV-infected women in our study is consistent with previous reports from Bahir Dar [9] and Addis Ababa [13], in which its prevalence among HIV infected individuals was 87.4 and 93.3%, respectively. In contrast, relatively low prevalence of *T. gondii* in HIV-infected individuals was documented in elsewhere [14]. This variability could be attributed to differences in climatic conditions and personal hygienic practices, and literacy status of the study subjects.

All IgM positive samples were also positive for IgG and lower rates of IgM seropositivity (2.2%) against *T. gondii* compared to IgG (94.4%) was observed in our study population. This finding is supported by other studies from India [15], Mexico [16] and South Africa [17] among HIV-infected individuals. The IgM antibody response to *Toxoplasma* infection is short-lived and it is frequently suppressed to undetectable levels in the case of severe immunocompromisation. Thus, the lower rates of detection of IgM antibodies in the routine diagnosis of toxoplasmosis in HIV-infected patients may be of limited value [6, 18]. Therefore, women with negative IgM test result shall not be taken as free from active infection and there should be further clinical investigation.

In this study, age was significantly associated with *T. gondii* seropositivity. Women within the age group between 28 and 37 years were 2.6 times more likely to be seropositive compared to those whose age was between 18 and 27 years [AOR = 2.58, 95% CI 1.01,6.60]. As age group of the study participants increases, a corresponding increases in *T. gondii* seropositivity. This is often mentioned by different scholars [10, 19]. This is probably related to prolonged exposure time as age increases.

The second significant predictor of *T. gondii* seropositivity in this study is educational status. Comparatively, the more educated participants had the less risk of *T. gondii* seropositivity and this finding was in accordance with a study done in Brazil in which participants with low educational status had higher prevalence of *T. gondii* antibody [20]. Participants with low educational status may have less hygienic practice and they are more likely to be exposed for the infection.

The other explanatory variable which showed significant association with seroprevalence of *T. gondii* was having substance abuse (chewing khat, alcohol drinking, and cigarette Smoking). This might be related with the habit of chewing khat (green leaf chewed for excitement) and it is known that khat addicted women masticate raw leaf without cooking. It is obvious that consumption of uncooked vegetables may expose peoples for acquisition of infections. Thus, chewing khat probably leads individuals to ingestion of the sporulated oocysts from of the parasite.

So far, a number of scholars stated that individuals having contact with cats have a significant association with seropositivity to anti-*T. gondii* IgG antibodies. Contradictory, our finding revealed that this variable is not significant risk factor of toxoplasmosis [9, 19]. Moreover, other behavioral risk factors such as; occupation, hand wash before meal, eating raw meat or vegetables, source of drinking water, and blood transfusion practice did not show significant association with seroprevalence of *T. gondii*. Being insignificant predictors of the above variables in this finding might be related with recall and social desirable biases of the participants. On top of this, extremely high prevalence of toxoplasmosis in our study may hide the nature of associations with its different risk factors.

## Conclusion

Seroprevalence of toxoplasmosis among the HIV-infected women in the childbearing age group in Mizan Aman was high. Age, educational status and drug addiction were identified as factors associated with *T. gondii* infection. Education of HIV-infected women about the transmission and prevention methods of *T. gondii* infection through health extension, in ART and antenatal care clinics is important. Further studies on incidence of toxoplasmosis in newborns and infants are recommended.

## Abbreviations

AIDS: acquired immune deficiency syndrome
AOR: adjusted odds ratio
ART: antiretroviral treatment
COR: crude odds ratio
ELISA: enzyme linked immune sorbent assay
HAART: highly active antiretroviral therapy
HIV: human immuno deficiency virus
IgG: immunoglobulin G
IgM: immunoglobulin M
TE: toxoplasmic encephalitis
